# The impact of Title IX iterations on campus sexual misconduct reports per synthetic control in the United States

**DOI:** 10.1057/s41271-025-00611-8

**Published:** 2025-12-01

**Authors:** Hannah Rochford, Corinne Peek-Asa, Whitney Zahnd, Keith Mueller, Brian Kaskie

**Affiliations:** 1 Department of Health Policy and Management, School of Public Health, Texas A&M University, 212 Adriance Lab Road, Office 116, College Station, TX 77845 USA; 2 Office of Research Affairs, University of California San Diego, Gilman Parking Structure, 3100 Gilman Dr, La Jolla, CA 92093 USA; 3 Department of Health Management and Policy, College of Public Health, University of Iowa, 145 N. Riverside Drive, Iowa City, IA 52242 USA

**Keywords:** Sexual misconduct, Sexual harassment, Sexual assault, Sexual violence, Campus violence, Title IX

## Abstract

Title IX regulation changes’ impact on sexual misconduct (SM) reporting to the institutions of higher education (IHE) in the United States (US) remain poorly understood. To examine trends in the rates of SM reports submitted to each American institution’s Title IX Office, we applied a synthetic control. US IHE members of the American Association of Universities comprised the ‘treated’ group, and Canadian IHE members of the Major Regional Associations were used to create a counterfactual proxy. Marginally significant increases (*P* = 0.08) in reports followed the 2017 Title IX guidance change (+ 1.18, + 4.51 and + 2.24 reports per 1000 enrolled students in 2017–2018, 2018–2019 and 2019–2020, respectively), and a marginally significant decrease (− 5.23 reports per 1000 enrolled students in 2020–2021) in SM reports to Title IX offices followed the 2020 Title IX iterations. Reporting and response structures, like those specified in Title IX iterations, may influence rates of SM reporting.

**Supplementary Information:**

The online version contains supplementary material available at 10.1057/s41271-025-00611-8.

## Key messages

- The high prevalence and severe consequences of sexual misconduct victimization in university campus environments warrants research and policy priority.
- Submitting an institutional report is a necessary first step to accessing the campus supports and protections that mitigate some of the adverse effects of sexual misconduct. Therefore, understanding how various Title IX guidance and regulations have impacted rates of sexual misconduct reporting is critical to shaping future Title IX iterations (and other policies informing organizational responses to sexual misconduct) in a way that maximizes equity and minimizes harm.
- Title IX iterations may influence rates of sexual misconduct reporting to the United States institutions of higher education.

## Introduction

The prevalence and severity of sexual misconduct on university campuses have prompted increasing public concern. ‘Sexual misconduct’ is an encompassing term that refers to a continuum of harmful sexual behaviors including quid pro quo sexual violence and sexual assault (attempted and completed rape, nonconsensual sexual contact, dating and domestic violence), any behavior of a sexual nature performed without the consent of the other party, and behaviors that impose a threatening and/or intimidating effect on the person against whom such conduct is directed (stalking) [1].

In the United States (US), a 2015 Washington Post-Kaiser Family Foundation study estimated that of university students (ages 17–26) in attendance at some point in the last four years, 7% of men and 25% of women experienced unwanted sexual incidents [2]. University campus settings are often characterized by high rates of sexual misconduct given these settings over represent individuals between 18 and 24 years of age, an age cohort at particular risk for experiencing and perpetrating sexual misconduct [3]. Further, university campuses often have a high prevalence of environmental risk factors that are associated with an elevated risk of sexual misconduct (e.g., alcohol consumption, emphasis on social gatherings) [4]. Sexual misconduct victims experience adverse physical, mental, behavioral, reproductive, and financial health outcomes [5–7]. Affected individuals in higher education environments also often experience adverse academic outcomes including reduced ability to concentrate, lower grades, reduced class attendance and activity engagement, and greater risk for dropping courses or withdrawing from higher education entirely [6, 7].

A necessary first step for students to access the campus supports and protections that mitigate some of the adverse consequences mentioned above, particularly the academic consequences, is submitting a report on the sexual misconduct experienced to the institution’s Title IX office. Their purpose is to ensure sex-based discrimination (including sexual harassment and sexual violence) does not occur in educational programs that receive US federal funding. Rates of sexual misconduct reports to institutions of higher education are a consequential outcome as barriers to reporting create barriers to accessing these supports. Due to these barriers and other factors, more than ninety percent of sexual assault victims on college campuses do not report the assault to their institution [8].

**Fig. 1:**
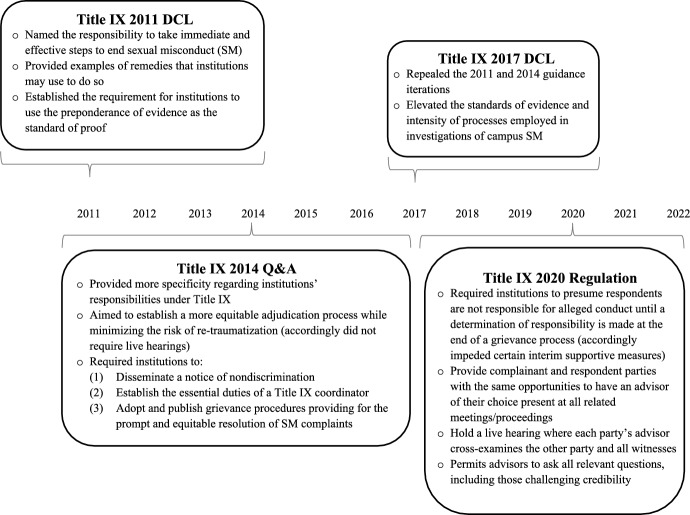
Title IX guidance/regulation changes related to sexual misconduct, 2011–2020

Under Title IX of the U.S. Education Amendments of 1972, federally funded institutions of higher education are liable for the negative academic effects resulting from sexual misconduct [9]. In response, Title IX and other sexual misconduct-related policies, such as the Jeanne Clery Disclosure of Campus Security Policy and Campus Crime Statistics Act, or Clery Act (1990), the Violence Against Women Act (1994, and subsequent reauthorizations) and the Campus Sexual Violence Elimination, or CampuSaVE Act (a 2013 amendment to the Clery Act) [10] have created strengthened expectations for how higher education institutions report, prevent and respond to instances of campus sexual misconduct [11]. The U.S. Department of Education (DOE) ensures that Title IX is upheld and issues recurrent changes to the guidelines that specify how Title IX is to be operationalized. Implementing Title IX necessitates achieving a challenging balance between three distinct and often competing interests in sexual misconduct investigations: (a) institutional interests in avoiding liability, (b) complainant (the term for an alleged survivor in a Title IX adjudication process) interests and rights under Title IX, and (c) respondent (the term for an alleged perpetrator in a Title IX adjudication process) interests and right to due process [7, 12]. A period of considerable change has compounded preexisting Title IX-related implementation challenges, and heighted public awareness about university campus sexual misconduct. Figure 1 and Supplementary Material Part 1 provide additional details as to the implications of each of these guidance iterations.

Understanding the impact of current and previous Title IX iterations has been a challenge in part due to data limitations. Prior to 2011, most institutions were only collecting Clery Act compliance data. Evidence suggests this data are likely not a valid reflection of the rate of sexual misconduct incidence at a given institution [13, 14], and therefore would be inappropriate to use to evaluate the impact of related policy. Further, not all institutions maintain records of reports submitted to entities other than campus police. While many institutions conduct sexual assault climate surveys, these are not standardized and therefore do not permit aggregation across institutions. Only one empirical work exists assessing the relationship of the 2020 regulation with reporting rates, and outcomes. This is a state-specific work using of data on reports submitted to the Title IX offices of New York institutions of higher education [15]. The descriptive and longitudinal analyses used, however, do not allow for causal interpretations.

In this study we estimated the impact of the 2017 Title IX guidance and the 2020 Title IX regulation changes on rates of sexual misconduct reporting at American Association of Universities (AAU) institutions by applying a quasi-experimental design to a national sample of institutions.

## Data and methods

**Fig. 2:**
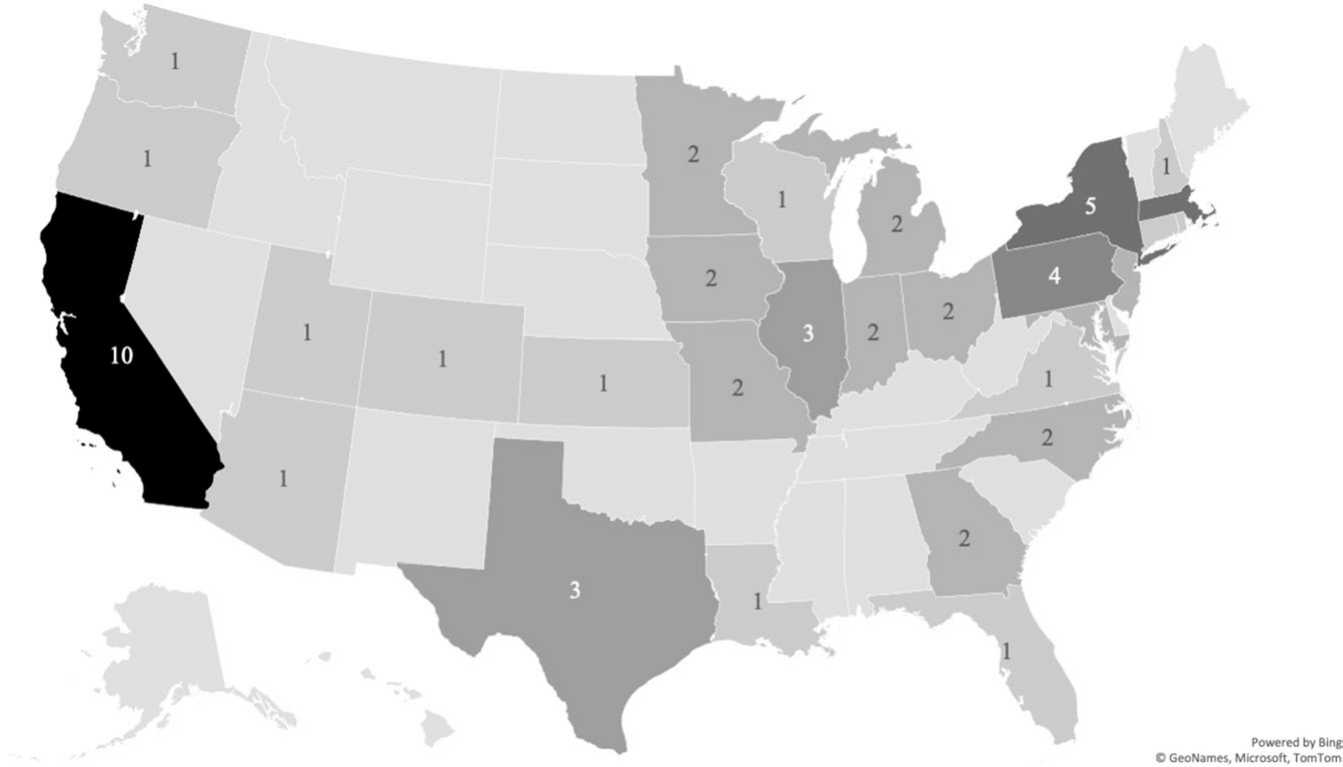
Map of Sampled Institution Locations

We sampled 64 U.S. institutions of higher education that are members of the AAU, an academic organization for leading research institutions. Of these 64 institutions, 29 are private and 35 are public. As of 2019, median fall student enrollment was 30,014, and the mean was 30,259. The upper decile of fall 2019 enrollment was 51,090, while the lower decile was 11,520. This sample of institutions is comprised those institutions of higher education with the greatest organizational capacity for ensuring compliance with complex regulations and maintaining records of sexual misconduct. These institutions span 30 different states, as shown in Fig. 2. This convenience sample was chosen to maximize the likelihood of institutions maintaining longitudinal records of Title IX reports, and in turn minimize dependency on imputed outcome values. While AAU institutions have greater organizational capacity for maintaining data of interest, these institutions do not necessarily hold sexual misconduct response as a greater or lesser priority than unsampled institutions.

### Outcome data

To collect rates of reports received by Title IX coordinators for sampled institutions, we manually abstracted data from public Title IX office reports, data received from requests made to each sampled institution, and student enrollment values from the National Center for Education Statistics (NCES). To improve the synthetic control fit, we applied the natural log of this measure. The acquisition of Title IX office data from the sampled institutions shown in the Supplementary Material Fig. S1.

***Publicly Released Data*** Colleges and universities may voluntarily release annual tallies of sexual misconduct reports received by their Title IX offices, though it is not federally required. Institutional web pages were consulted for data tools and/or reports specific to sexual misconduct cases addressed by their Title IX office. Twenty nine of the 64 schools made this data publicly available in at least some years of observation. Supplementary Material Part 1 specifies which institutional Title IX offices released this information and in which years. This data were manually abstracted.

***Non-Publicly Released Data*** Freedom of Information Act (FOIA) requests for the number of annual reports filed with the Title IX office within the period of interest were submitted to the 35 institutions that did not release this information publicly. To encourage cooperation, institutions that did not make this information publicly available were assured their identity would not be disclosed. Fourteen of the 35 cited their status as private institutions exempting them from FOIA requirements and declined to share the information. Ten replied that their institutions did not maintain the requested records. Of the eleven that maintained this data, four were able to contribute Title IX office data for the entire period. Four institutions were able to offer reports from 2015 forward, two were able to offer reports from 2016 forward, and one was able to offer reports from 2017 forward.

Of the 40 institutions included across seven years, 229 of the 280 data points were collected or abstracted. The remaining 51 (18.2%) were imputed using the process specified in Supplementary Material Part 1. The remaining 24 institutions from which no data could be collected were excluded from the final sample.

### Student population and explanatory data

The student population fall enrollment data for each institution from 2014 to 2021 was accessed from the National Center for Education Statistics.

***State-Level Controls*** The CDC [16] and existing literature highlight rates of general violence, and the economic and gender equity conditions individuals experience as risk factors for sexual misconduct [4, 17–19]. Relationships have also been established between risk for sexual misconduct and a community’s rate of alcohol consumption and social norms surrounding alcohol [20–23]. Norms pertaining to community tolerance for and sanctions against sexual misconduct are also a factor [19]. These concepts were controlled for using the measures summarized in Table 1. Because the period of interest overlaps with the height of the COVID-19 pandemic, the proportion of institutions with students enrolled fully online in Fall of 2020 (and therefore foreseeably less likely to encounter in-person forms of sexual misconduct) was also included.

**Table 1:** Explanatory variable summary

Explanatory variable summary			
Level of measure	Construct	Measure(s)	Source
State/province			
	Economic and gender equity conditions	Minimum wage	Bureau of labor statistics
		Unemployment rate	Bureau of labor statistics
		Female unemployment rate	Bureau of labor statistics
	Alcohol consumption	Binge drinking rate	Behavioral risk factor surveillance system
	Violence prevalence	UCR sexual assault rate	Federal bureau of investigation
	Community tolerance for sexual violence	#MeToo google trends	Google trendline
	Student presence	Proportion of students in only remote learning	National center for education statistics
Institution			
	Presence of Greek life	Binary indicator	Individual institution webpage
	University athletic Climate	Presence of division I athletics binary indicator	Individual institution webpage
		Presence of football, wrestling, hockey (0–3)	Individual institution webpage
	Climate survey use	Climate survey use	Individual institution webpage
	Urbanicity	RUCC	United States department of agriculture
	National news outlet incident	Binary indicator	Internet archive
	Institutional gender equity	Proportion of female professors	National center for education statistics
		Proportion of graduating undergraduate females	National center for education statistics

***Institution-Level Controls*** In campus settings specifically, the presence of certain social organizations are historically associated with nonconsensual sexual behavior [24–28]. Previous works also suggest that the urbanicity of an institution (population density surrounding the campus) may also account for variation in the campus contexts that institutions and survivors operate within [29]. Given the policies of interest here inform official institutional reporting and response processes, other factors indicating that an institution may be particularly attuned to this issue were included: whether or not the institutions has encountered national media attention related to sexual misconduct in the period of interest, whether or not the campus has independently conducted any sexual misconduct-related climate surveys in the period of interest, and institutional proportions of female faculty and of graduating female undergraduates. These concepts were controlled for using the measures summarized in Table 1.

### Data analysis

We applied synthetic control, a quasi-experimental design to assess the frequency with which Title IX iterations have been issued in the pre- and post-periods, about three academic years before and after. The pre-period for the 2017 Title IX iteration spans the 2014–2015, 2015–2016, and 2016–2017 academic years. The post-period for the 2017 Title IX iteration and the pre-period for the 2020 Title IX iteration spans the 2017–2018, 2018–2019, 2019–2020 academic years. The post-period for the 2020 Title IX iterations spans the 2020–2021, and 2021–2022 academic years. The details of designing synthetic controls and implementing the analysis are provided in Supplementary Material Part 2.

### Potential mechanisms for policy impact

There are three mechanisms underlying changes post- each Title IX iteration: one resulting in increased rates of sexual misconduct reporting, one resulting in no change, and one resulting in decreased reporting. With respect to the former, institutional efforts to educate their campus on the implications of each Title IX change may have made reporting individuals more aware of where and how to report, conferring a positive impact on reporting. With respect to the second, it is also reasonable to suspect that reporting individuals in higher education campus settings may not be attentive to either the policy changes or campus education efforts. This would result in no measurable effect on reporting. Finally, it could be argued that the public discourse regarding concerns and critiques of each Title IX iteration and/or institutional efforts to educate their campus on the implications of the change may have intimidated or otherwise deterred reporting individuals, which resulted in a negative impact on reporting.

## Results

### 2017 Title IX guidance

**Fig. 3:**
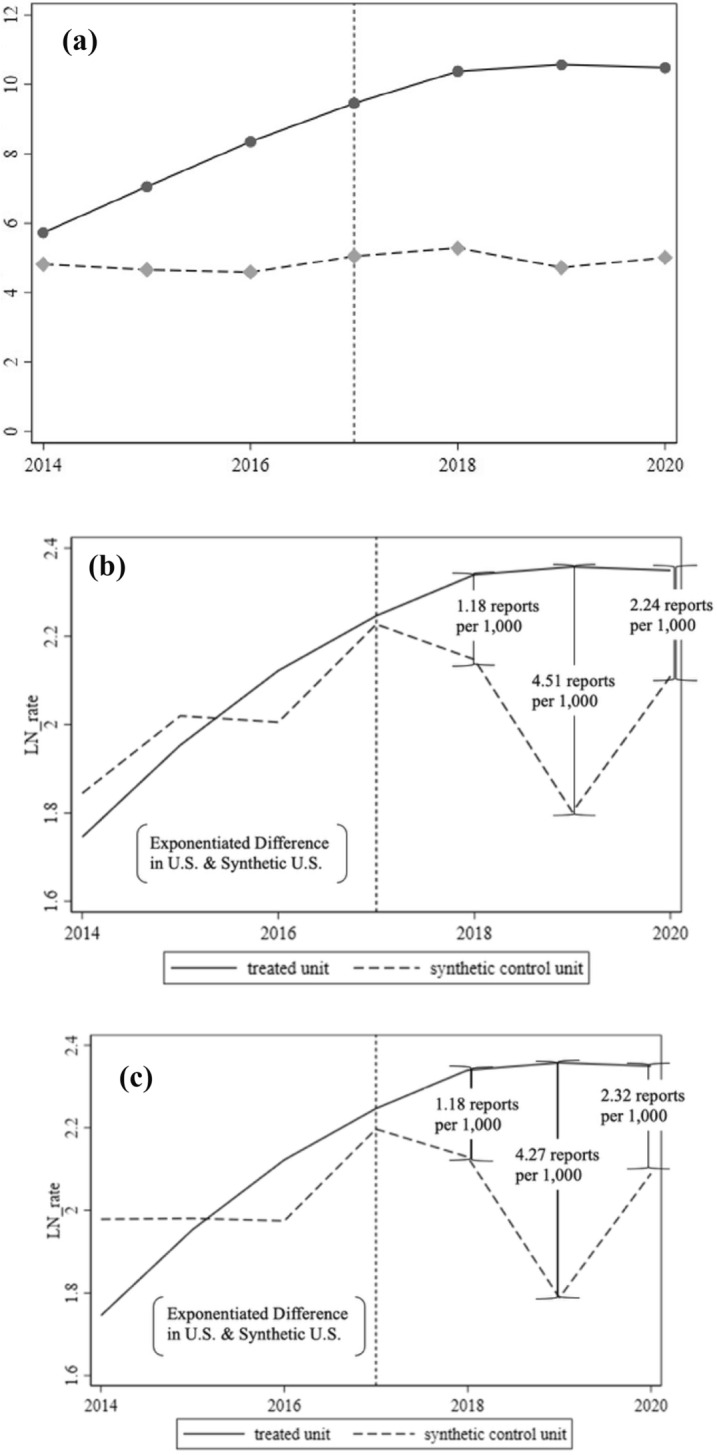
Longitudinal trends and synthetic control results | sexual misconduct per 1000 enrolled students at included U.S. and Canadian institutions, academic year 2014–2015 to academic year 2020–2021. **a** Sexual misconduct reports received per 1000 enrolled students at included U.S. institutions (solid, *n* = 40) and Canada institutions (dashed, *n* = 11). **b** Synthetic control for log-transformed reports of sexual misconduct per 1000 enrolled students, academic year 2014–2015 to academic year 2020–2021 for U.S. AAU institutions (solid) and synthetic U.S. institution (dashed); **c** bias-corrected synthetic control for log-transformed reports of sexual misconduct for U.S. AAU institutions (solid) and synthetic U.S. institution (dashed)

Our reports per 1000 enrolled students for participating institutions over the period of interest is displayed graphically in Fig. 3a. Applying the synthetic control approach to the natural log of reports to Title IX coordinator offices, we found an increase in the reports received by U.S. institutions after the 2017 regulations by slightly over one report per 1000 enrolled students at AAU institutions one year after implementation, slightly over four reports per 1000 enrolled students at AAU institutions after two years of implementation, and slightly over two reports per 1000 enrolled students at AAU institutions in the third year of implementation, as displayed in Fig. 3b. The bias-corrected synthetic control results were very stable relative to the original 2017 Title IX analysis, as indicated by Fig. 3c. This model suggests an additional 1.18, 4.27, and 2.32 reports per 1000 enrolled students at AAU institutions in the 2017–2018, 2018–2019, and 2019–2020 academic years, respectively. All other inference tables and figures derived using the biased-corrected synthetic control approach are available in Supplementary Material Part 2.

Table S2 summarizes the weights assigned to donor pool units to reach the synthetic control, suggesting the ‘synthetic US’ with the closest fit to the observed US trajectory in the pre-period was constructed by assigning 2.5% weight to donor institution one, 27.4% to donor institution three, 55.7% to donor institution five, 7.2% to donor institutions eight and nine, and 0% weight to all other institutions, summing to 100%.

Table S3 contrasts the predictor values for each explanatory variable included in the model. Comparable values between the affected unit, or the unit affected by a policy change, and the synthetic unit implies a strong ‘fit’, supporting the notion that it is appropriate to use the synthetic unit as a counterfactual proxy. Pearson tests confirm strong agreement between the affected unit and synthetic unit across most of our controls.

**Fig. 4:**
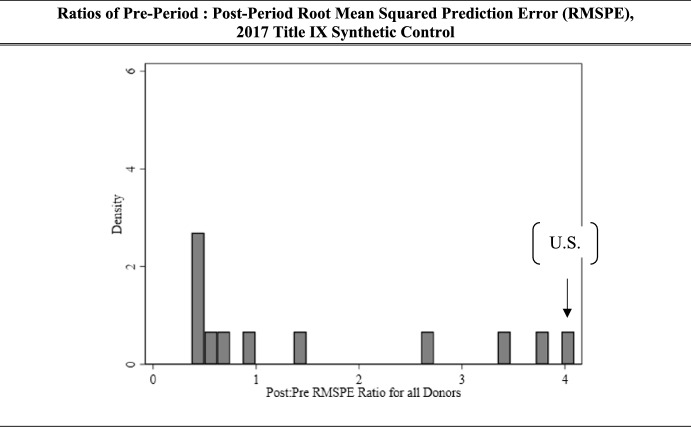
Ratios of pre-period: post-period root mean squared prediction error (RMSPE), 2017 Title IX synthetic control

Performing placebo tests in which we pretend each of our unaffected synthetic control donors were actually the affected group, we reach the ratios of pre-period and post-period RMSPE values displayed in Fig. 4. If a policy is impacting the outcome of interest, we would expect none of the pre:post ratios to be as extreme as our affected group, and that most of the placebo ratio values would be near one, which is what we found.

Ranking our ratio values from greatest to least, we converted this finding into a p-value, as displayed in Table 2. If we assume the Title IX 2017 guidance had no effect on rates of reporting, or a null of 0, we have would expect a result as extreme or more extreme than what we have observed here about 8% of the time. This is marginally significant, near but not reaching the alpha = 0.05 standard for significance.

**Table 2:** *P*-values for pre: post root mean squared prediction error, 2017 Title IX synthetic control

Unit	*P*-value for pre: post RMPSE
U.S. AAU institutions	**0.083**
Donor institution 1	0.500
Donor institution 2	0.750
Donor institution 3	0.417
Donor institution 4	0.167
Donor institution 5	0.584
Donor institution 6	0.999
Donor institution 7	0.667
Donor institution 8	0.833
Donor institution 9	0.917
Donor institution 10	0.334
Donor institution 11	0.250

**Fig. 5:**
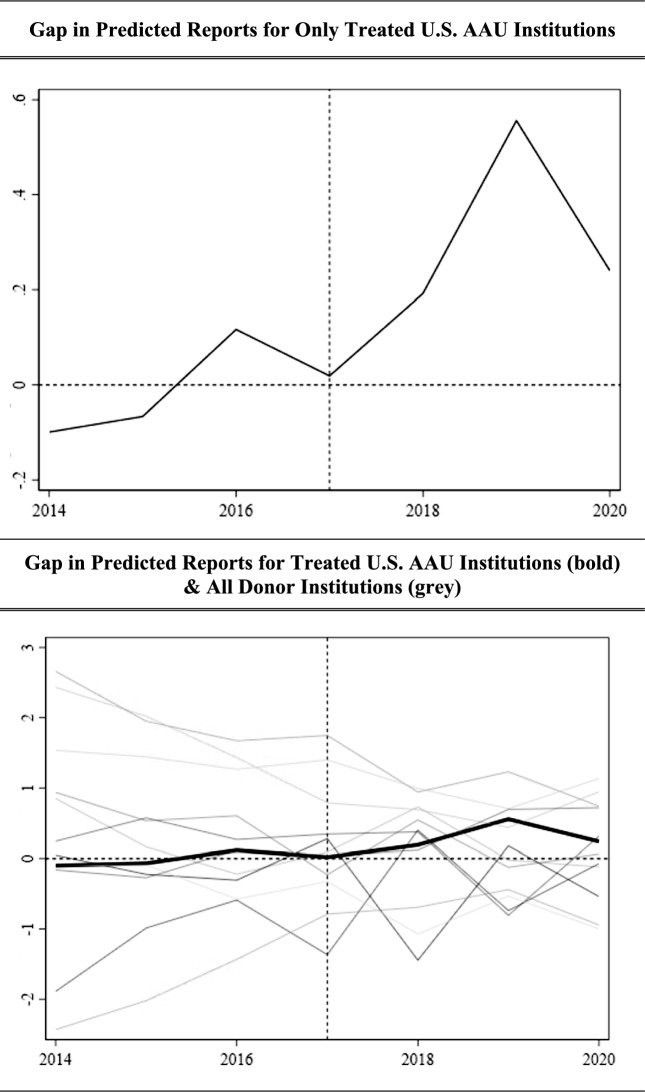
Gap in predicted reports of sexual misconduct per 1000 enrolled students pre- and post-2017 Title IX guidance, academic year 2014–2015 to academic year 2020–2021

A graph of the gap between our actual reports and our predicted report values for our affected and placebo units can be found in Fig. 5 (next page). Our treatment group is bolded, hovering near zero in the pre-period, and taking a notable dip in the post-period, whereas the gaps between our actual and predicted reports in both the pre- and post-periods follow much different paths for the unaffected placebo units.

### 2020 Title IX regulation

**Fig. 6:**
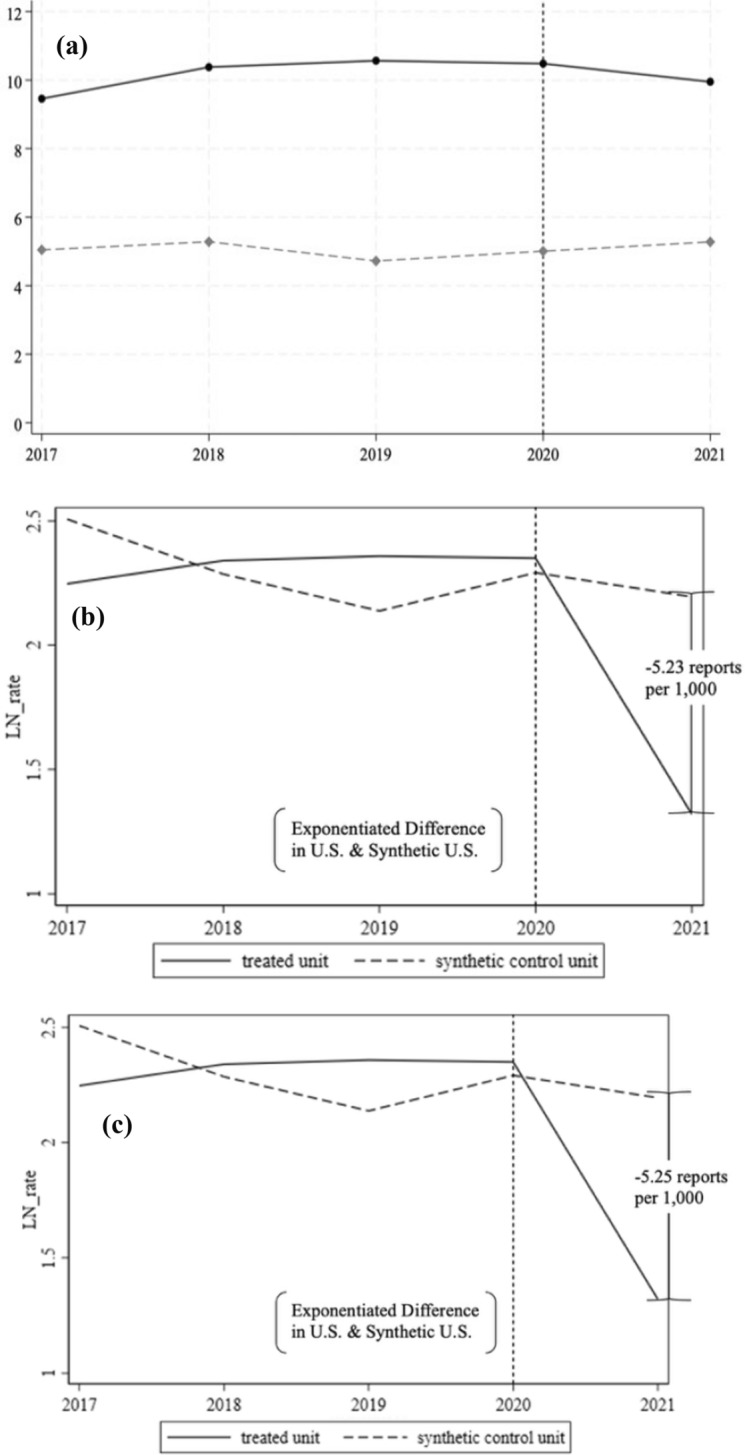
Longitudinal Trends and Synthetic Control Results | Sexual Misconduct per 1000 Enrolled Students at included U.S. and Canadian Institutions, academic year 2017–2018 to academic year 2021–2022. **a** Sexual misconduct reports received per 1000 enrolled students at included U.S. institutions (solid, *n* = 40) and Canada institutions (dashed, *n* = 11). **b** Synthetic control for log-transformed reports of sexual misconduct per 1000 enrolled students, academic year 2017–2018 to academic year 2021–2022 for U.S. AAU institutions (solid) and synthetic U.S. Institution (dashed). **c** Bias-corrected synthetic control for log-transformed reports of sexual misconduct for U.S. AAU institutions (solid) and synthetic U.S. institution (dashed)

Our reports per 1000 enrolled students for participating institutions over the period of interest are displayed graphically in Fig. 6a. Applying the synthetic control approach to the natural log of reports made to Title IX coordinator offices, we found an estimated decrease in the reports received by U.S. AAU institutions after the 2020 regulations of about five reports per 1000 enrolled students at AAU institutions, as displayed in Fig. 6b. The bias-corrected synthetic control results were very stable relative to the original 2020 Title IX analysis, as is indicated by Fig. 6c. This model similarly implies a decrease in reporting to Title IX Coordinator offices by approximately five per 1000 at AAU institutions. Applying the synthetic control approach iteratively to each individual U.S. institution, instead of treating the U.S. average as a single affected unit, we calculated a similar but slightly larger estimate of a 6.19 report decrease per 1000 enrolled students at AAU institutions. All other inference tables and figures derived using the biased-corrected synthetic control approach are available in Supplementary Material Part 3.

The weights assigned to donor pool units to reach the synthetic control are displayed in Supplementary Material Table S6 and suggest the ‘synthetic US’ with the closest fit to the observed US trajectory in the pre-period was constructed by assigning 16.3% weight to donor institution three, 6.8% to donor institution four, 76.9% to donor institution five, and 0% weight to all other institutions, summing to 100%. Table S7 contrasts the predictor values for each explanatory variable included in the model. Relatively strong agreement is again observed between the affected unit and synthetic unit across most controls.

**Fig. 7:**
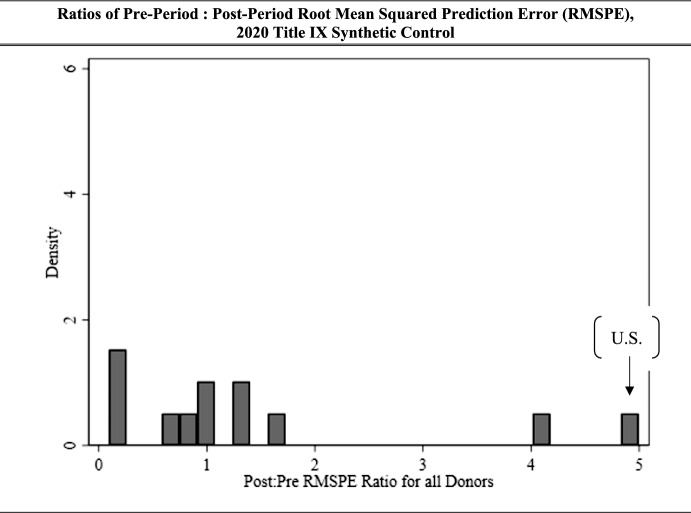
Ratios of pre-period: post-period root mean squared prediction error (RMSPE), 2020 Title IX synthetic control

Figure 7 displays the ratios of pre-period and post-period RMSPE values from performing placebo tests. We again find the pre: post ratios for the affected value are extreme relative to most of the ratios near one held by the donor units.

Ranking our ratio values from greatest to least, we converted this finding into a *p*-value, as displayed in Table 3. If we assume the Title IX 2020 regulation had no effect on rates of reporting, or a null of 0, we would expect a result as extreme or more extreme than what we had observed here about 8% of the time. This is marginally significant, near but not reaching the alpha = 0.05 standard for significance.

**Table 3:** *P*-values for pre: post root mean squared prediction error, 2020 Title IX synthetic control

Unit	*P*-value for pre: post RMSPE
U.S. AAU institutions	**0.083**
Donor institution 1	0.583
Donor institution 2	0.167
Donor institution 3	0.500
Donor institution 4	0.416
Donor institution 5	0.334
Donor institution 6	0.667
Donor institution 7	0.583
Donor institution 8	0.999
Donor institution 9	0.917
Donor institution 10	0.750
Donor institution 11	0.250

**Fig. 8:**
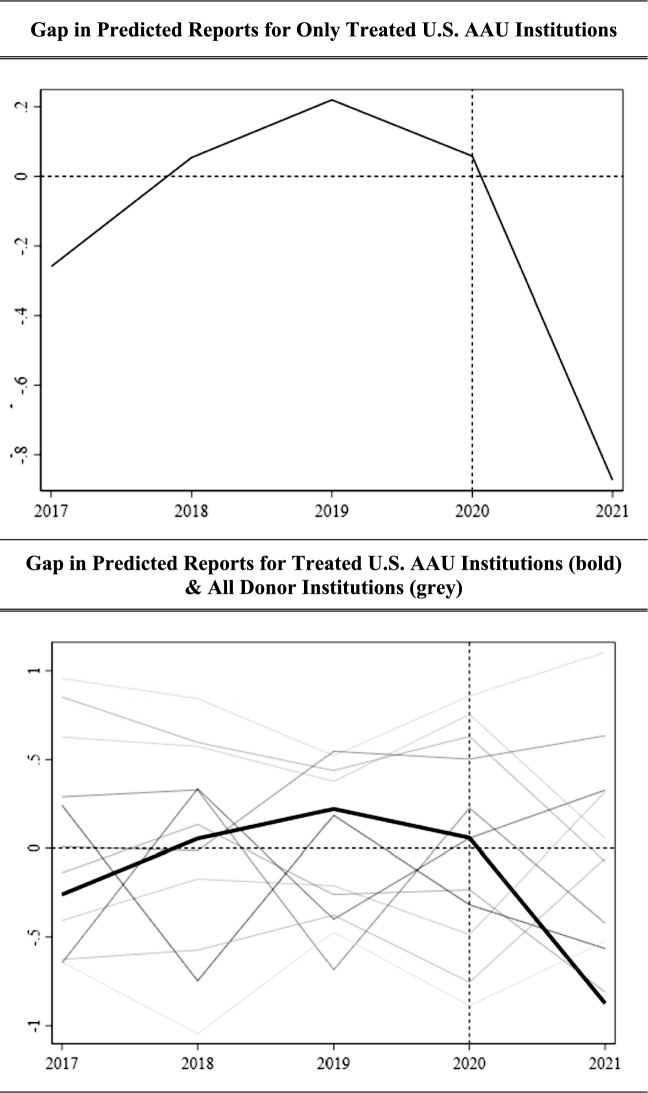
Gap in predicted reports of sexual misconduct per 1000 enrolled students pre- and post-2020 Title IX guidance, academic year 2017–2018 to academic year 2021–2022

Graphing the gap between our actual reports and our predicted report values for our affected and placebo units, we have Fig. 8 (next page). Our treatment group is bolded, hovering near zero in the pre-period, and taking a notable dip in the post-period, whereas the gaps between our actual and predicted reports in both the pre- and post-periods followed much different paths for the unaffected placebo units.

## Discussion

Our findings suggest a marginally significant increase in reports following the 2017 Title IX guidance change, and a marginally significant decrease in sexual misconduct reports to Title IX offices following the 2020 Title IX regulation change. These results suggest that the Title IX iterative changes guiding institutional policies and processes for response may impact rates of sexual misconduct reporting to AAU institutions of higher education. The trends associated with 2017 change suggest the first potential mechanism, wherein institutional efforts to educate their campus on the implications of each Title IX change may have made reporting individuals more aware of where and how to report, conferring a positive impact on reporting. Conversely, the 2020 results suggest the third potential mechanism was active following the 2020 Title IX regulation change, wherein public discourse regarding concerns and critiques of each Title IX iteration and/or institutional efforts to educate their campus on the implications of the change may have intimidated or otherwise deterred reporting individuals. Supplementary Material Part 4 places findings in context of other qualitative literature, and discusses: implications for sexual misconduct data infrastructure, intersections with other related policies (i.e., the Violence Against Women Act, the Cleary Act), the importance of continued research and evaluation, and of stability in expectations for IHE responses to sexual misconduct.

### Limitations

Findings offered here should be interpreted in the context of several limitations. Arguably the greatest of these is the very finite period of pre- and post-duration available to assess each policy. While additional years of observations before and after each change would strengthen the conclusions offered here, the synthetic control approach offers a unique way to derive a strong counterfactual, and in turn a policy impact estimate. Further, both the 2017 and 2020 Title IX changes co-occurred with other exogenous shocks: the #MeToo movement in October of 2017, and the COVID-19 pandemic in March of 2020. While controls for these factors were included in both analyses, and the impact of these shocks were felt by treated and untreated institutions, it is difficult to be certain that the estimates offered here are fully attributable to the policy changes. While only 7 of the observations included in the 2020 analysis were imputed (3.5%), a significantly larger portion of data in the 2017 analysis was imputed (51 or 25.5% of all observations, 44 of which were in the pre-period). While missingness indicators were used to verify imputation did not induce any outliers. Graphic analyses were used to verify post-imputation distributions resembled pre-imputation distributions. Despite these safeguards it is possible that the changes estimated (particularly in the 2017 analysis) are diluted relative to the underlying change because the pre-period imputations for the 2017 analysis are based in part on post-period observations for treated institutions. Third, the limited sample of included in this work undermines the external validity of these findings. AAU institutions were included here per their unique administrative capacities and financial resources available to collect and report these data. These and other differences between AAU and non-AAU institutions may render very different experiences under the same policy changes. The patterns of sexual misconduct reports made to more teaching focused or smaller institutions may not be reflected by the figures extended by the sampled institutions.

While the included institutions are representative of the larger AAU sample on many observable characteristics, it is possible that including additional AAU institutions would have added robustness to the findings. There is potential for systematic differences between institutions that held data and those that lacked data to contribute, and between institutions that shared data and those that were exempt from sharing related data. Anonymity was promised to institutions that did not make Title IX data publicly available to help manage participation hesitancy, but data from only about a third of the institutions that did not make Title IX data public was ultimately collected.

The sampled AAU institutions disproportionately represent those operating in California, New York, Massachusetts, Pennsylvania, Illinois, and Texas, and entirely exclude 22 states. It is therefore possible these findings reflect the intersection of these policy changes with other state-specific influences (fallout from highly public cases of sexual misconduct as in California, Massachusetts, and Texas, and/or state-specific policy changes as in New York).

Reporting and response structures, like those specified in Title IX iterations, may influence rates of SM reporting. Accordingly, additional research exploring the extent to which the impacts observed here generalize to other types of IHE settings and to IHE settings beyond the US are warranted.

## Conclusions

Our findings highlight the capacity for Title IX to either exacerbate or mitigate challenges with incident underreporting. We found marginally significant increases in reporting associated with the 2017 Title IX guidance change, and marginally significant decreases in reporting associated with the 2020 Title IX regulation change. This is consequential as submitting a report is a necessary first step to accessing many of institutional supports and educational opportunity protections. Any barriers to reporting then are also barriers to accessing these supports. Shaping the policy landscape that informs how institutions respond to reports of sexual misconduct in a way that facilitates reporting is a necessary initial step for advancing safety and equity in university and college campus settings.

## Supplementary Information

Below is the link to the electronic supplementary material.

Supplementary file1 (DOCX 183 KB)

Supplementary file2 (DOCX 451 KB)

Supplementary file3 (DOCX 465 KB)

Supplementary file4 (DOCX 31 KB)

## Data Availability

10.1080/07448481.2025.2461606.
