# Supplementary material for: The impact of Title IX iterations on campus sexual misconduct reports per synthetic control in the United States
Source: J Public Health Policy. 2025 Dec 1;47(1):40–59. doi: 10.1057/s41271-025-00611-8 (PMC13008768; doi:10.1057/s41271-025-00611-8)
Supplement: Supplementary file 1 — Supplementary file1 (DOCX 183 KB) [file 41271_2025_611_MOESM1_ESM.docx]

**Part I**

*Summary of Title IX Guidance/Regulation, 2011-2020*

On April 4, 2011, the U.S. Department of Education (DOE) Office for Civil Rights (OCR) issued the 2011 Dear Colleague Letter (DCL) addressing Title IX enforcement (1), and synthesizing previous guidance into a single, comprehensive document (1). The letter discussed Title IX requirements for responding to student-on-student sexual harassment, named college and university responsibilities in taking immediate and effective steps to end sexual harassment and sexual violence on college campuses (1), and provided examples of remedies that schools may use to prevent sexual harassment, and address its effects (1). The letter also established the requirement for universities and colleges to use the preponderance of the evidence as the standard of proof, requiring a showing that an event is more likely to have occurred than not, when determining whether sexual misconduct had occurred (1), which was significant given previous OCR guidance had not designated a standard evidentiary standard (1).

Responding to the need for additional guidance, the OCR issued the 2014 Questions & Answers (Q & A) document to provide more information regarding educational institutional responsibilities under Title IX. The guidance outlined three key procedural requirements: (1) disseminate a notice of nondiscrimination, (2) establish the essential duties of a Title IX coordinator, and (3) mandate the adoption and publication of grievance procedures that provide for the prompt and equitable resolution of student sexual misconduct complaints (1). With respect to the latter, the OCR specified that grievance procedures include: (1) an adequate, reliable, and impartial investigation of complaints, with opportunities for the respondent and complainant to present evidence and witnesses, (2) specified, reasonably prompt time frames for major stages in the complaint process, (3) written notice of the complaint outcome to the complainant and respondent, and (4) assurance that the school would take preventive steps to avoid the recurrence of any sexual misconduct and remedy any discriminatory effects on involved parties if appropriate (1). The aim of this guidance was to establish a more equitable adjudication process that did not require live hearings while ensuring that survivors of sexual misconduct could report and navigate the adjudication process without enduring additional trauma. In lieu of cross-examination, the DOE recommended indirect questioning, in which parties submit questions to a trained hearing panel to determine the relevance of and ask these questions (2). This guidance also permitted single investigator models in which complainants and respondents would entirely forego a hearing and adjudication would be completed using written exchanges (1).

The OCR then issued the 2017 Dear Colleague Letter to explicitly rescind the 2011 DCL and the 2014 Q & A guidance, and the 2017 Questions & Answers document to provide interim guidance on September 22, 2017. The 2017 DCL stated that the procedures for resolving allegations of sexual misconduct formed under previous guidance lacked “the most basic elements of fairness and due process, are overwhelmingly stacked against the accused, and are in no way required by Title IX law or regulation.” (1) Interim guidance required institutions to apply an evidence standard or a clear and convincing evidence standard to reach findings, make any opportunities and rights available to one party in an investigation equally available to the other party (1), and issue written notice to the responding party of the allegations constituting a potential violation of the prevailing institutional sexual misconduct policy, including sufficient details and with sufficient time to prepare a response before any initial interview. Despite the dissemination of interim guidance, higher education institutions found themselves in a state of limbo as to how to formulate their Title IX policies and procedures due in part to the rescission of previous guidance documents and due to imminent new proposed regulations by the Department of Education (DOE) (1).

**Figure S1.** Flow Chart of Title IX Office Data Acquisition


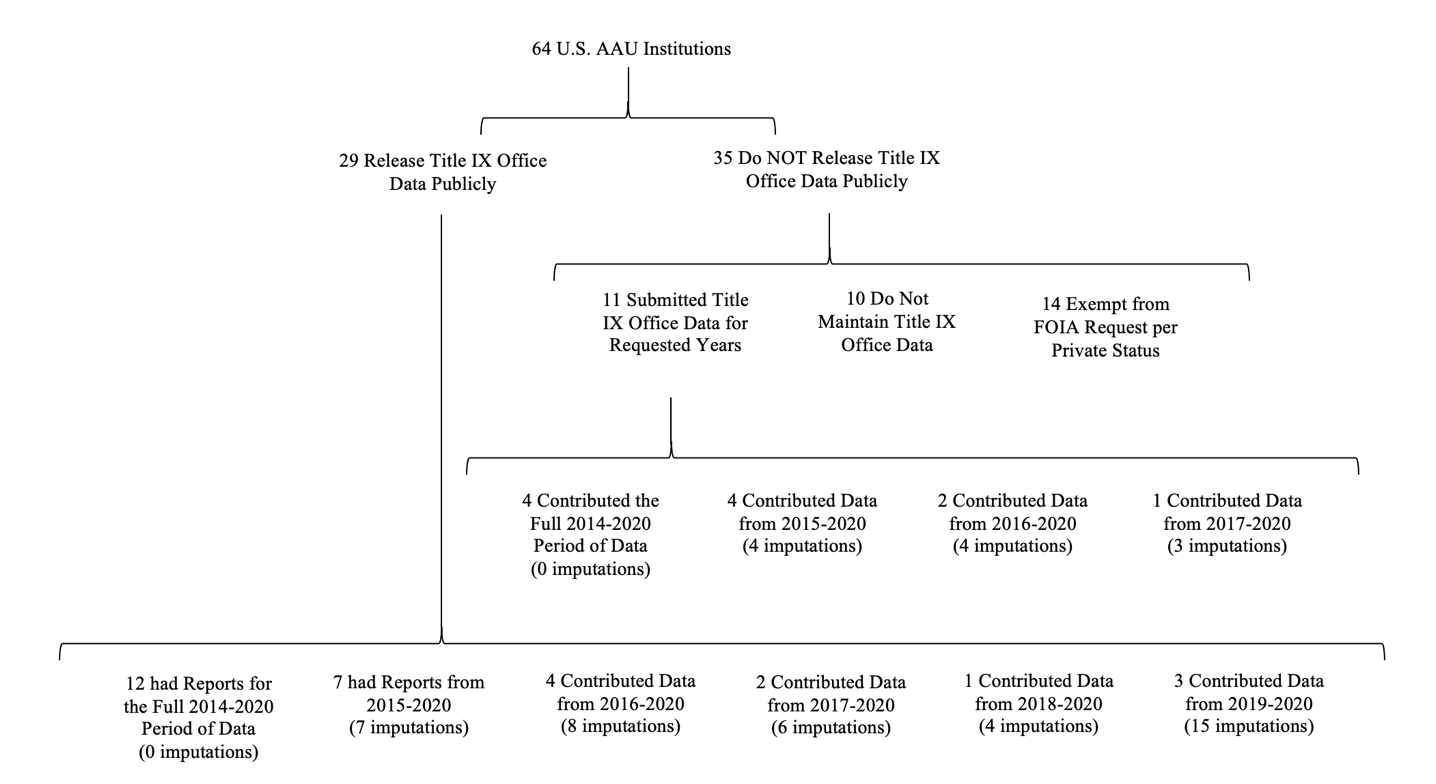


*Imputed Outcome Data*

***Publicly Released Data:*** Of the 29 institutions that released some Title IX office data publicly, 12 made available data from the full 2014-2020 period. Of the 17 institutions that did not have the full 2014-2020 period available, seven shared public Title IX office data spanning back to 2015 (requiring 1 imputation each), 4 institutions spanned back to 2016 (requiring 2 imputations each), 2 institutions spanned back to 2017 (requiring 3 imputations each), and 1 institution spanned back to 2018 (requiring 4 imputations), 3 institutions spanned back to 2019 (requiring 5 imputations each).

Of the 203 institution-year observations within the public data sample, 40 (19.7%) were imputed using STATA’s Poisson imputation function:

*mi set mlong*

*gen IMP = 0*

*replace IMP = 1 if reports == .*

*mi register imputed reports*

*mi impute poisson reports, add(1) by(unitid) rseed(20) force.*

**Figure S2** displays the proportion of data points imputed within each year included in the analysis.

**Figure S2.** Proportion of Outcome Data Imputed by Year

| **Proportion of Outcome Data Imputed by Year** |
| --- |
|  |

As discussed in (3) and (4), this parametric imputation method assumes an underlying Poisson distribution when imputing missing values of a count variable. It is based on the asymptotic approximation of the posterior predictive distribution of the missing data, or the trend line tangent to the curve predicting the missing data’s distribution. This method is appropriate here given (1) it assumes that missingness is not at random, and (2) ensures imputations are based in part on observed data from the institution itself.

***Non-Publicly Released Data:***  The four institutions that were able to contribute Title IX office data since 2015 each required 1 imputation. The two institutions that contributed Title IX office data since 2016 each required 2 imputations. The institution that contributed Title IX data from 2017 forward required 3 imputations.

Of the 77 institution-year observations within the non-public data sample, 11 (14.3%) were imputed using the same STATA 17.0’s Poisson imputation function as above.

**References**

1. Wiseman S. RE-TOOLING TITLE IX: HOW ADOPTING INTERMEDIARY CROSS-EXAMINATION IN TITLE IX SEXUAL MISCONDUCT ADJUDICATION CAN PROVIDE FAIRNESS AND DUE PROCESS FOR ALL. Univ Louisville Law Rev. 2020;59(1):125–68.

2. Holland K, Bedera N, Webermann A. The Selective Shield of Due Process: Analysis of the U.S. Department of Education’s 2020 Title IX Regulations on Live Cross-Examination. Anal Soc Issues Public Policy. 2020;20(1):584–612.

3. Raghunathan T, Lepkowski J, Hoewyk J, Solenberger P. A Multivariate Technique for Multiply Imputing Missing Values Using a Sequence of Regression Models. Surv Methodol. 2000 Nov 30;27.

4. van Buuren S. Multiple imputation of discrete and continuous data by fully conditional specification. Stat Methods Med Res. 2007 June 1;16(3):219–42.
