# Supplementary material for: The impact of Title IX iterations on campus sexual misconduct reports per synthetic control in the United States
Source: J Public Health Policy. 2025 Dec 1;47(1):40–59. doi: 10.1057/s41271-025-00611-8 (PMC13008768; doi:10.1057/s41271-025-00611-8)
Supplement: Supplementary file 2 — Supplementary file2 (DOCX 451 KB) [file 41271_2025_611_MOESM2_ESM.docx]

**Part 2**

*Synthetic Control*

Quasi-experimental designs are meant to offer an empirically defensible estimate of what would have happened in the event the jurisdiction of interest never changed the policy being assessed. This ‘what would have happened’ concept is referred to as the ‘counterfactual’. Here, all U.S. institutions of higher education experience each of the Title IX guidance or regulation changes concurrently. Therefore, to generate a counterfactual proxy we considered institutions in other nations that had parallel processes for sexual misconduct reporting and response, but were unaffected by the Title IX changes of the last decade. Included Canadian institutions of higher education with membership in the Major Regional Associations held nearly equivalent definitions of sexual misconduct, pathways for reporting, processes for investigating and adjudicating instances of sexual misconduct, and mediation alternatives that parallel the informal resolution pathways available under Title IX at U.S. institutions. These definitions and processes were verified by institution-specific process documents and clarifying conversations with institutional leaders tasked with preventing and responding to sexual misconduct. Neither nation imposes federal requirements for related data to be maintained nor released publicly, therefore only a portion of the sampled Canadian institutions held and elected to release available data. Of the 56 Canadian Institutions within Major Regional Associations, which offers us a parallel sampling pool to the U.S. AAU, 11 institutions maintained this data and shared related annual counts.

The frequency with which Title IX iterations have been issued constrains the pre- and post-periods available to assess each, about three academic years before and after. The pre-period for the 2017 Title IX iteration spans the 2014-2015, 2015-2016, and 2016-2017 academic years. The post-period for the 2017 Title IX iteration and the pre-period for the 2020 Title IX iteration spans the 2017-2018, 2018-2019, 2019-2020 academic years. The post-period for the 2020 Title IX iterations spans the 2020-2021, and 2021-2022 academic years.

Leveraging the finite variation available is necessary to estimate the impacts of 2017 and 2020 Title IX iterations. A combination of donor pool units may approximate the characteristics of the affected unit better than a single unaffected unit would alone. That is the core notion of the synthetic control approach. Rather than relying on the parallel trends of an existing control group to estimate our counterfactual, we manufacture one. Using data from donor pool institutions from Canada that were unaffected by the Title IX policy changes of interest, we create a ‘synthetic U.S.’, or a weighted average of units in the donor pool that closely resemble our affected unit in the pre-period. The set of non-negative weights sum to one and are assigned such that the difference between the unit affected by the policy and the synthetic comparator are minimized within the pre-intervention period with outcome variable predictors. This same weighted average is extended to the outcome variable predictors in the post-period. Our policy impact estimate is derived from the difference between the group outcomes of our synthetic control and treatment groups in the post-intervention period.

While synthetic control techniques now exist for assessing instances with multiple-intervention units (1), these require longer pre-periods than what the frequency of Title IX policy changes make available. Therefore, in addition to applying the classic synthetic control approach using the average of all included U.S. institutional sexual misconduct reports per 1,000 enrolled students as a single intervention unit, we applied two sensitivity analyses. First, we applied a bias-corrected variant of the synthetic control approach on this U.S. average outcome (2). Second, we manually apply the synthetic control approach to each of the 40 U.S. institutions affected by the Title IX policy changes individually, and derived an average estimate from this series of analyses.

*Inference Tests*

A series of inference tests were performed within each of these analyses. First, we generated a graphic of the gap between the predicted and observed outcome in the pre- and post-periods. If the policy of interest has impacted the outcome, we expect to have a gap near zero in the pre-period, and a gap away from zero in the post-period. Next, by iteratively treating each donor unit as though it were the unit affected by the policy of interest within the synthetic control, we generated a distribution of placebo effects. We applied this distribution graphically by plotting the gap of the placebos with the gap of our affected group. The placebo distributions also allowed us to calculate the Root Mean Square Prediction Error (RMSPE) for each placebo in the pre- and post-periods, then compute the ratio of these values. If the policy of interest impacted the outcome, we expect to have the ratio of our affected unit be greater than one (larger after change RMSPE than before change RMSPE), and those of the placebo units to be near one (comparable RMSPE values in the before and after change periods). If the policy of interest did not impacted the outcome, we expect the affected unit to also have a ratio near one.

Sorting the ratios from greatest to least allowed us to confirm the affected unit with the most extreme ratio. Further, it allowed us to generate a *p*-value indicating the probability of encountering a result as extreme or more extreme than the observed value given a policy that had no effect. The smaller the *p*-value, the more likely it is that a relationship between the policy and outcome exists.

*2017 Supplemental Results*

**Table S2.** Canadian Institution Donor Weights, 2017 Title IX

| **Canadian Institution Donor Weights,**  **Synthetic Control** | | | | **Canadian Institution Donor Weights,**  **Bias-Corrected Synthetic Control** | | | |  |
| --- | --- | --- | --- | --- | --- | --- | --- | --- |
|  |  | | | |  |  | |  |
| Donor Institution 1 | | 0.025 | Donor Institution 1 | | | | 0.025 | |
| Donor Institution 2 | | 0 | Donor Institution 2 | | | | 0 | |
| Donor Institution 3 | | 0.274 | Donor Institution 3 | | | | 0.274 | |
| Donor Institution 4 | | 0 | Donor Institution 4 | | | | 0 | |
| Donor Institution 5 | | 0.557 | Donor Institution 5 | | | | 0.557 | |
| Donor Institution 6 | | 0 | Donor Institution 6 | | | | 0 | |
| Donor Institution 7 | | 0 | Donor Institution 7 | | | | 0 | |
| Donor Institution 8 | | 0.072 | Donor Institution 8 | | | | 0.072 | |
| Donor Institution 9 | | 0.072 | Donor Institution 9 | | | | 0.072 | |
| Donor Institution 10 | | 0 | Donor Institution 10 | | | | 0 | |
| Donor Institution 11 | | 0 | Donor Institution 11 | | | | 0 | |
|  |  | | | |  |  | |  |

**Table S3**. Predictor Balance, 2017 Title IX Synthetic Control

| **Sexual Misconduct Reports Received by Title IX Offices per Student** | | | |
| --- | --- | --- | --- |
| Level of Measure | Predictor | Treated Unit | Synthetic Unit |
|  |  |  |  |
| State / Province |  |  |  |
|  | Minimum Wage | 8.37 | 12.66 |
|  | Female Unemployment Rate | 5.16 | 6.38 |
|  | Unemployment Rate | 4.83 | 6.85 |
|  | Binge Drinking Rate | 0.16 | 0.25 |
|  | UCR Sexual Assault Rate | 41.54 | 59.99 |
|  | #MeToo Google Trends | 90.00 | 71.57 |
|  | Proportion of Students in only Remote Learning | 0.61 | 0.53 |
| Institution |  |  |  |
|  | Presence of Greek Life | 1.00 | 0.98 |
|  | Presence of Division I Athletics | 0.91 | 1.00 |
|  | Football, Wrestling, Hockey (0-3) | 1.32 | 1.68 |
|  | Climate Survey Use | 0.57 | 0.44 |
|  | RUCC | 1.59 | 1.60 |
|  | National News Outlet Incident | 0.34 | 0.14 |
|  | Proportion of Female Professors | 0.41 | 0.28 |
|  | Proportion of Graduating Undergraduate Females | 0.64 | 0.61 |
|  |  |  |  |
|  |  |  |  |

**Table S4.** Predictor Balance, 2017 Title IX Bias-Corrected Synthetic Control

| **Sexual Misconduct Reports Received by Title IX Offices per Student** | | | |
| --- | --- | --- | --- |
| Level of Measure | Predictor | Treated Unit | Synthetic Unit |
|  |  |  |  |
| State / Province |  |  |  |
|  | Minimum Wage | 8.91 | 12.66 |
|  | Female Unemployment Rate | 5.04 | 6.08 |
|  | Unemployment Rate | 4.44 | 6.44 |
|  | Binge Drinking Rate | 0.16 | 0.25 |
|  | UCR Sexual Assault Rate | 41.54 | 70.70 |
|  | #MeToo Google Trends | 90.00 | 71.57 |
|  | Proportion of Students in only Remote Learning | 0.61 | 0.53 |
| Institution |  |  |  |
|  | Presence of Greek Life | 1.00 | 0.93 |
|  | Presence of Division I Athletics | 0.91 | 1.00 |
|  | Football, Wrestling, Hockey (0-3) | 1.31 | 1.76 |
|  | Climate Survey Use | 0.57 | 0.16 |
|  | RUCC | 1.55 | 1.40 |
|  | National News Outlet Incident | 0.33 | 0.07 |
|  | Proportion of Female Professors | 0.42 | 0.30 |
|  | Proportion of Graduating Undergraduate Females | 0.64 | 0.60 |
|  |  |  |  |
|  |  |  |  |

**Figure S3.** Gap in Predicted Reports of Sexual Misconduct per 1,000 Enrolled Students Pre- and Post-2017 Title IX Guidance per Bias-Corrected Synthetic Control, Academic Year 2017-2018 – Academic Year 2021-2022

| **Gap in Predicted Reports for Treated U.S. Institutions** | **Gap in Predicted Reports for Treated U.S. Institutions (bold)**  **& All Donor Institutions (grey)** |
| --- | --- |
| 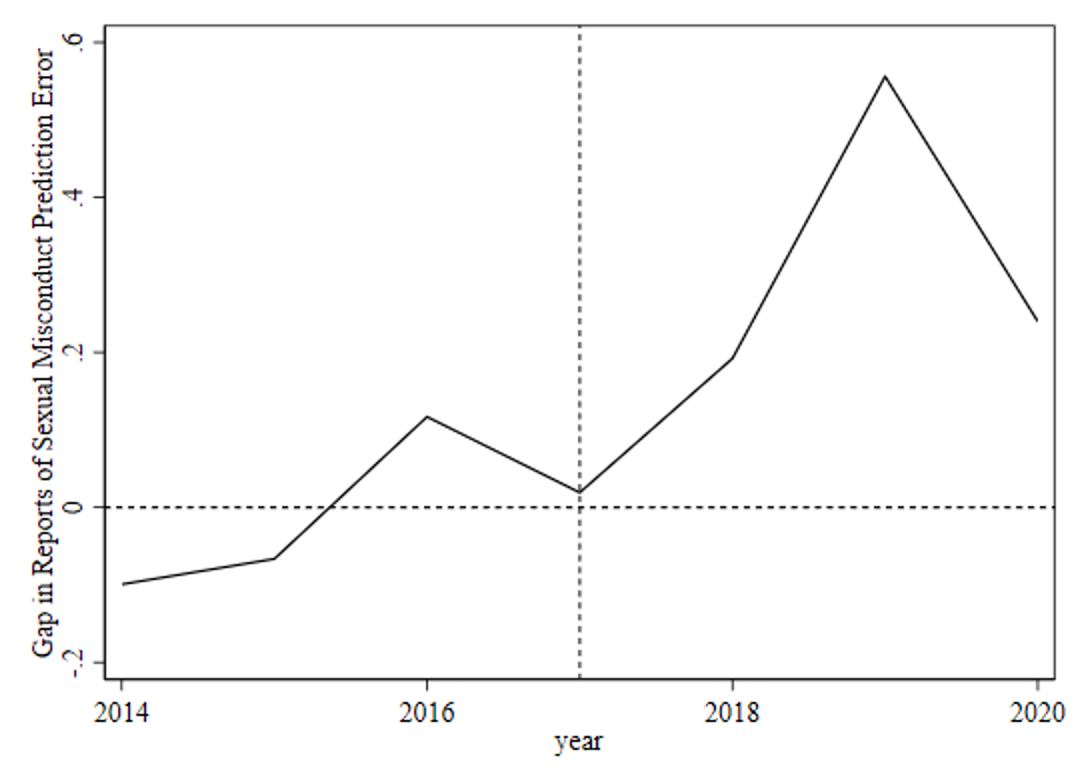 | 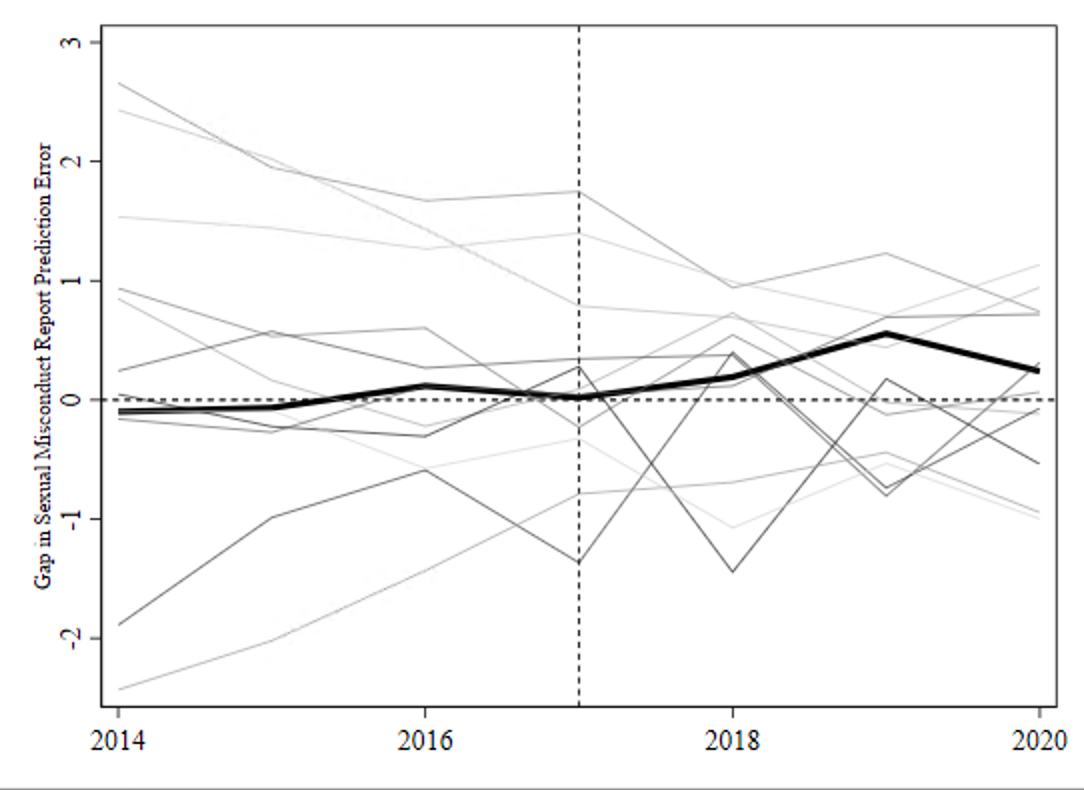 |
|  | |

**Figure S4.** Ratios of Pre-Period : Post-Period Root Mean Squared Prediction Error (RMSPE), 2017 Title IX Bias Corrected Synthetic Control

| **Ratios of Pre-Period : Post-Period Root Mean Squared Prediction Error (RMSPE),**  **2017 Title IX Bias-Corrected Synthetic Control** |
| --- |
| 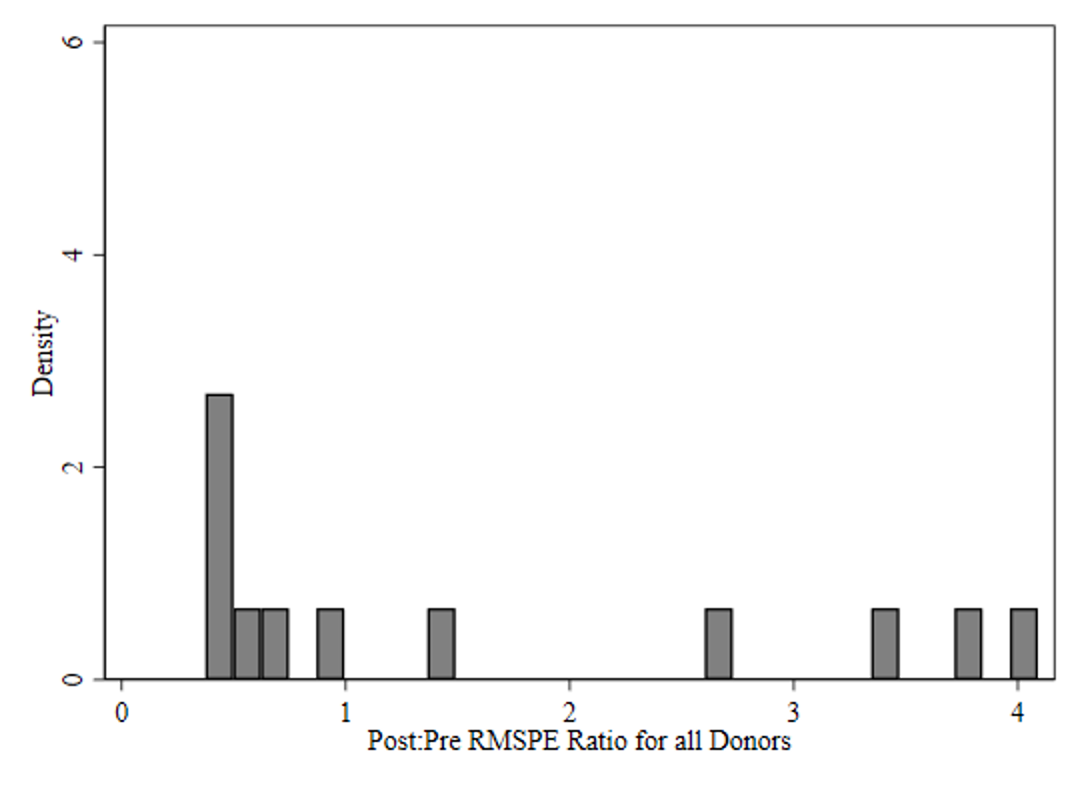  U.S. |
|  |

**Table S5.** p-Values for Pre : Post Root Mean Squared Prediction Error, 2017 Title IX Bias-Corrected Synthetic Control

| **Unit** | **p-value for Pre : Post RMSPE** |
| --- | --- |
|  |  |
| U.S. Institutions | **0.083** |
| Donor Institution 1 | 0.500 |
| Donor Institution 2 | 0.750 |
| Donor Institution 3 | 0.417 |
| Donor Institution 4 | 0.167 |
| Donor Institution 5 | 0.584 |
| Donor Institution 6 | 0.999 |
| Donor Institution 7 | 0.667 |
| Donor Institution 8 | 0.833 |
| Donor Institution 9 | 0.917 |
| Donor Institution 10 | 0.334 |
| Donor Institution 11 | 0.250 |
|  |  |

*Single-Institution Average Synthetic Control*

Applying the synthetic control approach iteratively to each individual U.S. institution, instead of treating the U.S. average as a single affected unit, we found similar estimates of 1.19, 5.68, and 3.74 more reports per 1,000 students enrolled at AAU institutions than what would have been expected per our synthetic control in the 2017-2018, 2018-2019, and 2019-2020 academic years.

**References**

1. Xu Y. Generalized Synthetic Control Method: Causal Inference with Interactive Fixed Effects Models. Polit Anal. 2017 Jan;25(1):57–76.

2. Wiltshire JC. allsynth: Synthetic Control Bias-Correction Utilities for Stata. 2021 Stata Conf. 2022;
