# Supplementary material for: The impact of Title IX iterations on campus sexual misconduct reports per synthetic control in the United States
Source: J Public Health Policy. 2025 Dec 1;47(1):40–59. doi: 10.1057/s41271-025-00611-8 (PMC13008768; doi:10.1057/s41271-025-00611-8)
Supplement: Supplementary file 3 — Supplementary file3 (DOCX 465 KB) [file 41271_2025_611_MOESM3_ESM.docx]

**Part 3**

*2020 Supplemental Results*

**Table S6.** Canadian Institution Donor Weights, 2020 Title IX

| **Canadian Institution Donor Weights,**  **Synthetic Control** | | | | **Canadian Institution Donor Weights,**  **Bias-Corrected Synthetic Control** | | | |  |
| --- | --- | --- | --- | --- | --- | --- | --- | --- |
|  |  | | | |  |  | |  |
| Donor Institution 1 | | 0 | Donor Institution 1 | | | | 0 | |
| Donor Institution 2 | | 0 | Donor Institution 2 | | | | 0 | |
| Donor Institution 3 | | 0.163 | Donor Institution 3 | | | | 0.163 | |
| Donor Institution 4 | | 0.068 | Donor Institution 4 | | | | 0.068 | |
| Donor Institution 5 | | 0.769 | Donor Institution 5 | | | | 0.769 | |
| Donor Institution 6 | | 0 | Donor Institution 6 | | | | 0 | |
| Donor Institution 7 | | 0 | Donor Institution 7 | | | | 0 | |
| Donor Institution 8 | | 0 | Donor Institution 8 | | | | 0 | |
| Donor Institution 9 | | 0 | Donor Institution 9 | | | | 0 | |
| Donor Institution 10 | | 0 | Donor Institution 10 | | | | 0 | |
| Donor Institution 11 | | 0 | Donor Institution 11 | | | | 0 | |
|  |  | | | |  |  | |  |

**Table S7**. Predictor Balance, 2020 Title IX Synthetic Control

| **Sexual Misconduct Reports Received by Title IX Offices per Student** | | | |
| --- | --- | --- | --- |
| Level of Measure | Predictor | Treated Unit | Synthetic Unit |
|  |  |  |  |
| State / Province |  |  |  |
|  | Minimum Wage | 8.91 | 12.66 |
|  | Female Unemployment Rate | 5.04 | 6.08 |
|  | Unemployment Rate | 4.44 | 6.44 |
|  | Binge Drinking Rate | 0.16 | 0.25 |
|  | UCR Sexual Assault Rate | 41.54 | 70.70 |
|  | #MeToo Google Trends | 132.8 | 107 |
|  | Proportion of Students in only Remote Learning | 0.315 | 0.29 |
| Institution |  |  |  |
|  | Presence of Greek Life | 1.00 | 0.93 |
|  | Presence of Division I Athletics | 0.91 | 1.00 |
|  | Football, Wrestling, Hockey (0-3) | 1.31 | 1.76 |
|  | Climate Survey Use | 0.57 | 0.16 |
|  | RUCC | 1.55 | 1.40 |
|  | National News Outlet Incident | 0.33 | 0.07 |
|  | Proportion of Female Professors | 0.42 | 0.30 |
|  | Proportion of Graduating Undergraduate Females | 0.64 | 0.60 |
|  |  |  |  |
|  |  |  |  |

**Table S8.** Predictor Balance, 2020 Title IX Bias-Corrected Synthetic Control

| **Sexual Misconduct Reports Received by Title IX Offices per Student** | | | |
| --- | --- | --- | --- |
| Level of Measure | Predictor | Treated Unit | Synthetic Unit |
|  |  |  |  |
| State / Province |  |  |  |
|  | Minimum Wage | 8.91 | 12.66 |
|  | Female Unemployment Rate | 5.04 | 6.08 |
|  | Unemployment Rate | 4.44 | 6.44 |
|  | Binge Drinking Rate | 0.16 | 0.25 |
|  | UCR Sexual Assault Rate | 41.54 | 70.70 |
|  | #MeToo Google Trends | 132.8 | 107 |
|  | Proportion of Students in only Remote Learning | 0.315 | 0.29 |
| Institution |  |  |  |
|  | Presence of Greek Life | 1.00 | 0.93 |
|  | Presence of Division I Athletics | 0.91 | 1.00 |
|  | Football, Wrestling, Hockey (0-3) | 1.31 | 1.76 |
|  | Climate Survey Use | 0.57 | 0.16 |
|  | RUCC | 1.55 | 1.40 |
|  | National News Outlet Incident | 0.33 | 0.07 |
|  | Proportion of Female Professors | 0.42 | 0.30 |
|  | Proportion of Graduating Undergraduate Females | 0.64 | 0.60 |
|  |  |  |  |
|  |  |  |  |

**Figure S5.** Gap in Predicted Reports of Sexual Misconduct per 1,000 Enrolled Students Pre- and Post-2020 Title IX Guidance per Bias-Corrected Synthetic Control, Academic Year 2017-2018 – Academic Year 2021-2022

| **Gap in Predicted Reports for Only Treated U.S. Institutions** | **Gap in Predicted Reports for Treated U.S. Institutions (bold)**  **& All Donor Institutions (grey)** |
| --- | --- |
| 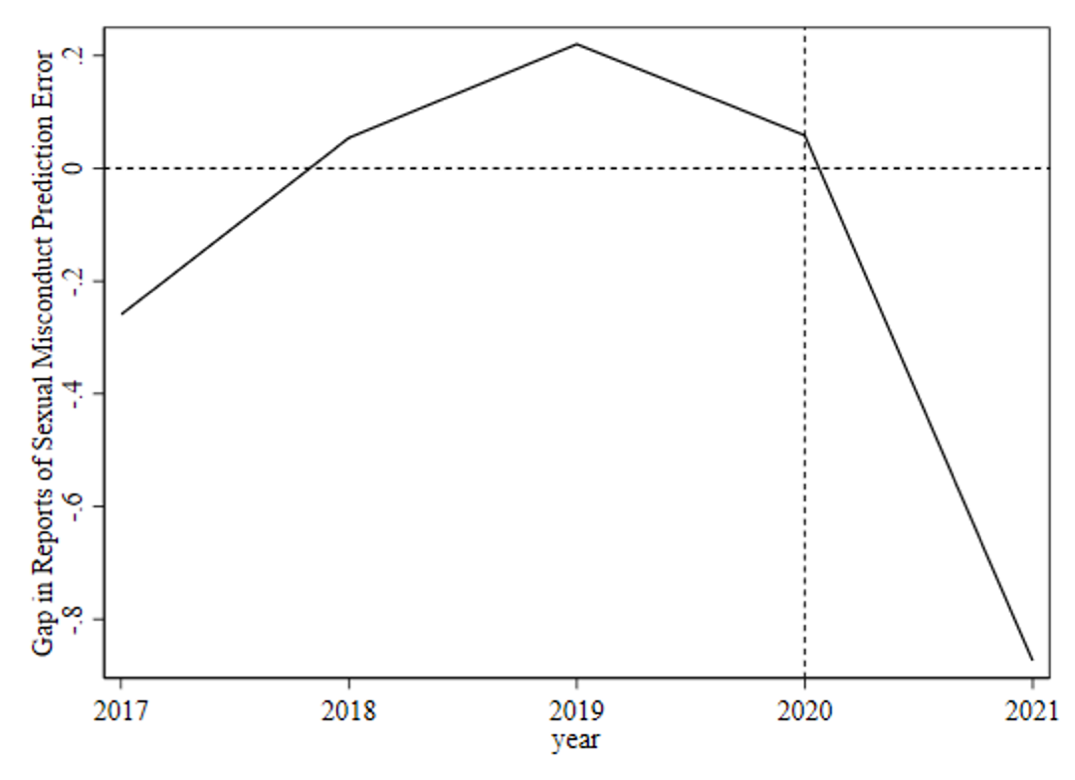 | 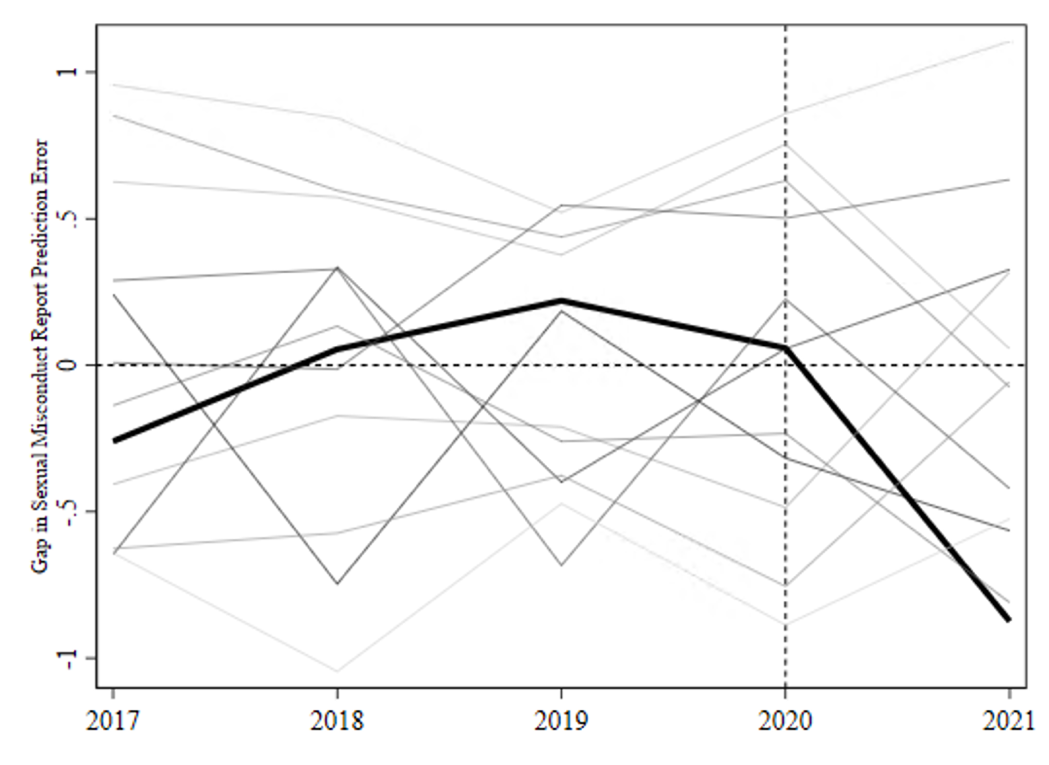 |
|  | |

**Figure S6.** Ratios of Pre-Period : Post-Period Root Mean Squared Prediction Error (RMSPE), 2020 Title IX Bias-Corrected Synthetic Control

| **Ratios of Pre-Period : Post-Period Root Mean Squared Prediction Error (RMSPE),**  **2020 Title IX Bias-Corrected Synthetic Control** |
| --- |
| 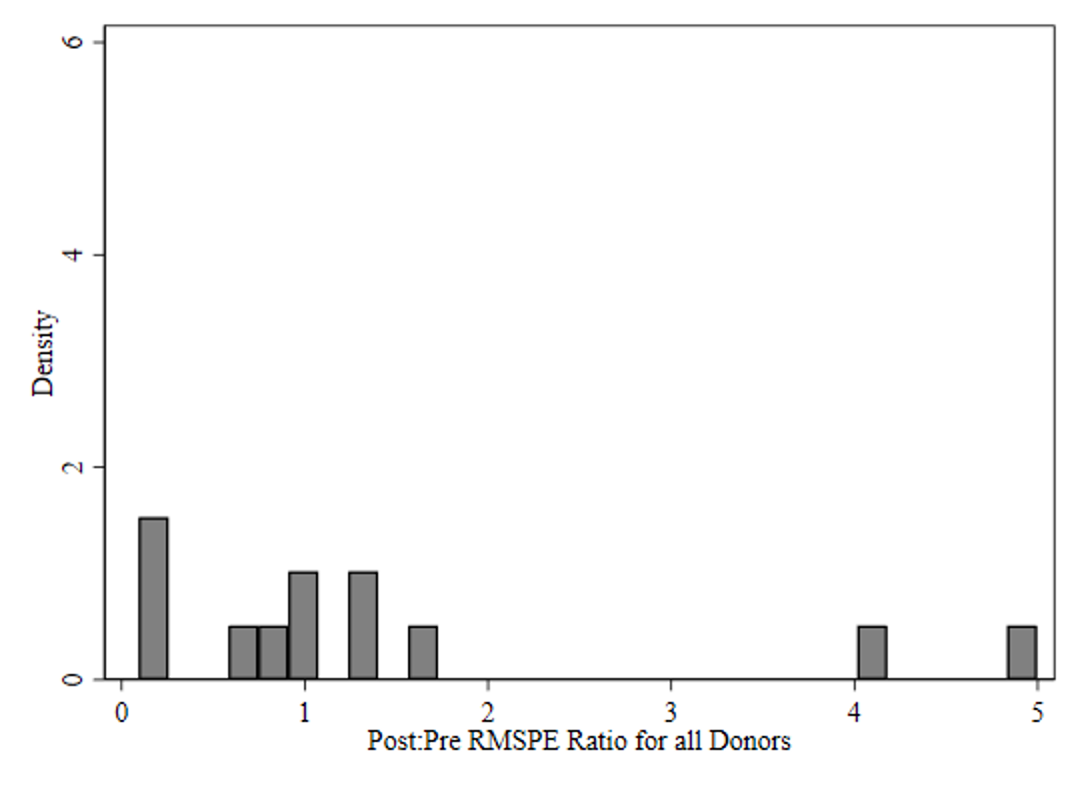  U.S. |
|  |

**Table S9.** P-Values for Pre : Post Root Mean Squared Prediction Error, 2020 Title IX Bias-Corrected Synthetic Control

| **Unit** | **p-value for Pre : Post RMSPE** |
| --- | --- |
|  |  |
| U.S. Institutions | **0.083** |
| Donor Institution 1 | 0.167 |
| Donor Institution 2 | 0.250 |
| Donor Institution 3 | 0.333 |
| Donor Institution 4 | 0.416 |
| Donor Institution 5 | 0.500 |
| Donor Institution 6 | 0.583 |
| Donor Institution 7 | 0.667 |
| Donor Institution 8 | 0.750 |
| Donor Institution 9 | 0.833 |
| Donor Institution 10 | 0.916 |
| Donor Institution 11 | 0.999 |
|  |  |
