# Supplementary material for: The impact of Title IX iterations on campus sexual misconduct reports per synthetic control in the United States
Source: J Public Health Policy. 2025 Dec 1;47(1):40–59. doi: 10.1057/s41271-025-00611-8 (PMC13008768; doi:10.1057/s41271-025-00611-8)
Supplement: Supplementary file 4 — Supplementary file4 (DOCX 31 KB) [file 41271_2025_611_MOESM4_ESM.docx]

**Part 4**

Other works offer qualitative insights into why these patterns may have emerged (1–5). These works synthesized observations that pre-2017 Title IX iterations aspired to advance gender equity, but generated criticism for compromising the due process rights of respondents (5). Recommendations in other works investigating Title IX iterations pre-2017 suggest that expanding restorative justice approaches like the informal resolutions added in Title IX 2020 may offer a crucial means for increasing fair outcomes in adjudication and relieving challenges with due process (2). Content analyses done on sexual misconduct policies from 2016-2017 suggest that many institutional policies acknowledged, but did not fully comply with Title IX requirements (4). This finding supports the notion that imposing regulation rather than guidance with the 2020 Title IX iteration may have heightened institutional implementation integrity. Works conducted specifically on the 2020 Title IX regulations noted concerns that the fear of institutional administrators of not being perceived as a “neutral” party can subtly perpetuate a system that marginalizes those who experience sexual misconduct (1).

The implications for institutions could include opportunities to identify campus-specific concerns or barriers related to reporting and take steps to manage these within the parameters established by Title IX. Federal policy makers have data that supports the notion that the provisions they imbed in the next iteration of Title IX can either increase or impede reporting rates.

Sustained evaluation efforts warrant priority as Title IX, and the campus sexual misconduct policy landscape more broadly, continue to evolve. If the absence of a strong evidence base remains, future regulatory iterations are vulnerable to the ideological leanings of public decision makers, and institutions of higher education will remain vulnerable to significant and frequent changes as has been observed throughout the last decade. Conducting robust evaluations of future Title IX regulatory and guidance iterations will allow the DOE to proactively identify patterns that indicate which institutions are not effectively conducting primary prevention, and/or are not effectively balancing the interests of involved parties as they respond to instances of sexual misconduct. Further, this would strengthen the evidence base describing which policy components advance the aims of maximizing equity and minimizing harm, and whish components have unintended consequences. The integration of evidence-based practices into both prevention and response processes can result in improved outcomes for involved parties and reduced institutional liability.

Using evidence from policy evaluations to isolate ideal approaches for prevention and response may also minimize the frequency and magnitude of changes to subsequent Title IX guidance and regulation. As campuses benefit from stable processes and liability reduction, less time and money will need to be expended on ensuring campus understanding of updated processes. This is a long-term benefit however, as institutions are on the verge of another Title IX regulation change as the newest proposed regulation was released in the summer of 2022, and final regulations take effect August 2024.

Making policy evaluations feasible will require improvements in the related data infrastructure. As was noted previously, institutions are not required to maintain, nor release data on reports submitted to Title IX offices. Therefore, the most readily available data on campus sexual misconduct are derived from the 2013 Violence Against Women Act call for institutional Clery Act-mandated annual fire and safety reports to include statistics on several forms of sexual misconduct (rape, statutory rape, incest, dating violence, domestic violence and stalking) reported to campus police (6). Previous works suggest that these figures are far from representative of the volume of sexual misconduct instances reported to institutional Title IX coordinator offices (7) and far from representative of the underlying prevalence of sexual misconduct on college campuses (8). A 2016 analysis of Clery Act data disclosed by 11,000 U.S. college and universities found that 89% had zero instances of rape reported to campus police, and 77% had zero instances of rape and zero instances of other forms sexual assault, fondling, domestic violence, dating violence and stalking reported to campus police (9). These data are therefore inappropriate for use in research related to campus sexual misconduct outcomes. While the most recent reauthorization of the Violence Against Women Act mandates campus wide sexual safety climate surveys at higher education institutions (H.R. 1620 (R.F.S), 117^th^ Congress), institutions will have the flexibility to develop and use their own surveys. Without standardizing what information is being collected across all institutions, it will remain difficult to develop a deep understanding of the implications of each new Title IX regulation.

Addressing this data gap could be accomplished by requiring Title IX offices to extend standardized reports with annual tallies of reports received, complaints made, and report/complaint outcomes to the DOE, and then making these figures available through the DOE’s Campus Crime and Safety Data tools. This would make significant progress in making rigorous analyses of Title IX guidance/regulation possible. Many institutions already volunteer this information, and their leaders could be invited as partners in determining what the standardized reports should include.

*Future Research*

Repeating the 2020 Title IX analysis as data from the 2022-2023 academic year become available data will be valuable given there will have been a longer implementation period, and the outcome data from this year will reflect a period of normal levels of campus presence and interactions. As the sexual misconduct reporting data infrastructure across institutions improves, repeating these analyses with a larger and more diverse sample of institutions would be valuable given the limited number of included AAU institutions are rather homogenous. Expanded data availability would also make analyses within strata of institutions possible. Stratifying by key institutional differences and repeating both the 2017 and 2020 analyses would add clarity as to potential differences in policy impact between campuses. Some example differences might include private and public institution status, and whether an institution was already using a hearing model prior to the 2020 regulations, or if a single investigator approach was in place that would imply more dramatic institutional process changes were required to reach compliance with the 2020 regulations. Doing so would allow the estimates to reflect differences in institutional experiences with the policy changes per these or other characteristics.

Analyses of the outcomes of reports are also crucial to understanding if the policies of interest are advancing intended equity aims and appropriately balancing involved interests. After a report is made, the active iteration of Title IX shapes which recourse pathways are available to reporting parties, and what process each pathway requires. Modeling rates of uptake of each pathway by reporting individuals will offer insights as to what specific adjustments ought to be made to process requirements in future Title IX regulatory and guideline iterations.

**References**

1. Cruz J. The Constraints of Fear and Neutrality in Title IX Administrators’ Responses to Sexual Violence. J High Educ. 2021;92(3):363–84.

2. Harper S, Maskaly J, Kirkner A, Lorenz K. Enhancing Title IX Due Process Standards in Campus Sexual Assault Adjudication: Considering the Roles of Distributive, Procedural, and Restorative Justice. J Sch Violence. 2017 July 3;16(3):302–16.

3. Lorenz K, Hayes R, Jacobsen C. “Keeping the Wound Open”: Survivor Experiences with Title IX Investigations. Women Crim Justice. 2022;1–21.

4. Porter KB, Levitsky SR, Armstrong EA. Gender Equity and Due Process in Campus Sexual Assault Adjudication Procedures. J High Educ. 2022 June 15;1–27.

5. Sulfaro V, Gill R. Title IX: Help or Hindrance? J Women Polit Policy. 2019;40(1):204–27.

6. Hall CAB. Reporting Realities: Are the Clery Act and Other Federal Policies Failing Women at Institutions of Higher Education? [Internet] [Ph.D.]. The University of Texas at Arlington; 2020 [cited 2023 Jan 15]. Available from: https://www.proquest.com/docview/2472079491/abstract/55D4D6E9E19E4728PQ/1

7. Richards TN, Gillespie LK, Branscum C. Comparing sexual misconduct incidents in Annual Security Reports and Title IX coordinator data: Do policies increase reporting. J Am Coll Health. 2021 July 22;1–12.

8. Gardella JH, Nichols-Hadeed CA, Mastrocinque JM, Stone JT, Coates CA, Sly CJ, et al. Beyond Clery Act Statistics: A Closer Look at College Victimization Based on Self-Report Data. J Interpers Violence. 2015 Feb 1;30(4):640–58.

9. American Association of University Women. AAUW : Empowering Women Since 1881. 2022 [cited 2023 Jan 15]. An Underreported Problem: Campus Sexual Misconduct. Available from: https://www.aauw.org/resources/article/underreported-sexual-misconduct/
